# Crisis management: the perspectives of physicians working in family physician teams in Lithuania

**DOI:** 10.1017/S1463423622000615

**Published:** 2023-01-09

**Authors:** Aida Budrevičiūtė, Gediminas Raila, Renata Paukštaitienė, Leonas Valius

**Affiliations:** 1 Chief researcher of the biomedical study “Challenges of COVID-19 in Family Medicine”, Lithuania; 2 Lithuanian University of Health Sciences, Department of Family Medicine, Kaunas, Lithuania; 3 Lithuanian University of Health Sciences, Medical Academy, Department of Physics, Mathematics, and Biophysics, Kaunas, Lithuania

**Keywords:** COVID-19 pandemic, crisis management, family medicine, primary health care, Lithuania

## Abstract

**Aim:**

To assess the opinions of physicians working in family physician teams regarding COVID-19 (threat perception, overall work satisfaction, patient satisfaction with services provided, patient access to services, and the need for new tools for service provision).

**Methods:**

An anonymous survey of physicians (*N* = 191) working in family physician teams. Questionnaires were distributed among family physicians with the permission of the managers of their institutions and were collected by the lead researcher within 1–8 weeks. The quantitative study was conducted from 21 June 2021 to 17 September 2021. In total, 398 questionnaires were distributed, yielding a response rate of 48%, or 9% of the total population. Thirty-nine primary health care institutions (PHCIs) were randomly selected for the study: 11 public and 28 private.

**Findings:**

Older respondents and those with more years of work experience strongly agreed that the COVID-19 pandemic threatened their lives and safety, as well as that of their colleagues. Work satisfaction decreased during the pandemic among older respondents, those with more years of work experience, and those who had been employed at their current institution for longer. Respondents with more work experience believed that patient satisfaction with the services provided by their family medical institution decreased. Older respondents with more work experience asserted that patient access to services decreased during the pandemic. Physicians working further away from urban centers indicated a greater need for new tools in the effort to provide consultations compared to city-based physicians.

**Conclusions:**

The current health care crisis prompted by the COVID-19 pandemic is defined by the perception of threats to life and safety among physicians, an overall drop in their work satisfaction, decreased patient satisfaction with services provided, reduced patient access to services, and a greater need for new tools for providing consultations.

## Introduction

The COVID-19 pandemic prompted a crisis in the health care sector, which struggled to maintain the accessibility of services to patients; ensure patient satisfaction; promote the work satisfaction and well-being of physicians; and develop and apply new tools in remote patient care (Dominguez-Salas *et al*., 2021). Scientists recommend mitigating these challenges through effective communication and special training for medical professionals (Abideen *et al*., 2020). During the pandemic, physicians feared staff shortages; difficulties in the work environment, leadership, and management of their health care institution; and increased workload (Abd-Ellatif *et al*., 2021). Health care workers were under a staggering amount of stress and perceived COVID-19 as a threat to their lives and safety, which impacted their work satisfaction and could result in professional burnout (Dymecka *et al*., 2021). A survey of health care workers in Italy during the COVID-19 pandemic presented the following findings: 7.5% experienced symptoms of depression; 37.9% experienced psychological distress; 30.5% felt discriminated against; and 5.7% reported having experienced violence (Moro *et al*., 2022). Elsewhere, an estimated 16.5% of physicians experienced a sense of fear, which negatively impacted their work satisfaction (42%) (Abd-Ellatif *et al*., 2021). Another study found that elements of psychological stress – stress vulnerability, anxiety symptoms, loneliness, and irritability – had a negative impact on work satisfaction among health care workers, and loneliness was observed as having a significantly greater effect than other elements (Caponnetto *et al*., 2022). The following measures were implemented to manage the challenges posed by the COVID-19 pandemic: the establishment of a mentally safe work environment; the implementation of leadership programs; the introduction of measures increasing employee well-being and communication; and assistance provided to the health care team (Dominguez-Salas *et al*., 2021). One study established a link between family physicians’ work satisfaction and burnout syndrome, anxiety, and depression, which can potentially have negative consequences on work efficiency (Yilmaz, 2018). It was determined that during the pandemic physicians experienced increased levels of exhaustion and burnout, whereas nursing staff noted a drop in work satisfaction (Ruiz-Fernandez *et al*., 2020). Diabetes and hypertension remained the key reasons for visiting a family physician during the pandemic (Stephenson *et al*., 2021). Patient anxiety and depression were reportedly the main reasons for remote consultations with a family physician (90.6% of consultations), and disease prevention measures shrunk compared to pre-pandemic times (Stephenson *et al*., 2021). Patients faced reduced access to health care services during the pandemic, with the elderly, those with fewer technological resources, and those with lower health literacy being among the worst affected (Albert *et al*., 2021). Among primary health care specialists, 58% expressed concern over their patients’ limited ability to use certain technologies, and 55% were concerned with their patients’ overall technological knowledge (Mohammed *et al*., 2021). However, these findings also indicate that 74.5% of patients were satisfied with remote services provided by physicians (Mohammed *et al*., 2021). Novelty services (such as remote consultations) introduced over the course of the pandemic could certainly remain in use in the post-pandemic world (Albert *et al*., 2021). The key questions posed in this study are:

1. Does the perception of pandemic-related threats depend on respondents’ sociodemographic characteristics?

2. Did work satisfaction among physicians decrease during the COVID-19 pandemic?

3. How do physicians view the satisfaction of their patients with the provision of family health care services?

4. How did the pandemic affect the accessibility of services for patients?

5. Does the need for new tools for remote consultations depend on the locale of the respondent’s current workplace (urban/rural setting)?

## Methods

### Study design

Based on other studies, the researchers developed an instrument for a quantitative study (a questionnaire) and conducted a pilot study to test its validity. Upon implementing the necessary corrections, the main survey was conducted among physicians working in family physician teams. Permission to conduct the study was issued in advance by the Kaunas Regional Committee of Biomedical Research Ethics. The following hypotheses were postulated for interrogation via the study results:

H1: The perceptions of physicians regarding threats to their life and safety during the COVID-19 pandemic are statistically significantly dependent on the sociodemographic characteristics of the respondents.

H2: Work satisfaction among physicians decreased during the COVID-19 pandemic.

H3: Patient satisfaction with the services provided by the family medical institution decreased during the pandemic.

H4: Physicians perceived a decrease in their patients’ level of access to services during the pandemic.

H5: The opinions of respondents regarding the need for new tools for remote consultations during the COVID-19 pandemic depend on the location of their workplace (urban/rural setting).

### Source of data

A pilot study was conducted on 21–30 June 2021 involving 13 physicians: 3 from public PHCIs, 9 from private PCHIs, and 1 physician who worked in both public and private PHCIs. An anonymous survey of physicians (*N* = 191) working in family physician teams was carried out from 21 June 2021 to 17 September 2021. The respondents signed an informed consent form prior to participation. Of the 398 questionnaires distributed, 191 were used in the final analysis, and 4 were invalid, resulting in a response rate of 48% – that is, 9% of the sample population. A total of 39 PHCIs were randomly selected for the study; of these, 11 were public and 28 were private. The respondents were distributed as follows: 31% were employed by a private PHCI; 63% were employed by a public PHCI; and 6% worked for both. In terms of location, 88% of the respondents were city-based and 12% resided in rural areas. Females comprised 84% of respondents and males 16%. The regional distribution of the respondents was as follows: Vilnius county – 33%, Kaunas county – 18%, Klaipėda county – 8%, Šiauliai county – 15%, Panevėžys county – 13%, Tauragė county – 7%, and Telšiai county – 6%.

### Sample

The sample size was representative of the age, gender, and distribution of physicians in different counties in Lithuania. The 50/50 principle was applied when selecting respondents to ensure the participation of physicians from both public and private PHCIs. According to the data provided by the Institute of Hygiene, 1,903 family physicians and 238 internal medicine physicians were employed by primary health care institutions (PHCIs) and care homes at the end of 2020.

### Statistical analysis

IBM SPSS Statistics 27 was used for data analysis. The quantitative variables did not meet the conditions of normal distribution and were therefore compared in three groups using the nonparametric Kruskal–Wallis test and pairwise comparisons with the Bonferroni correction. The results were described in medians and quartiles (Q_1_–Q_3_). The relationship between qualitative variables was analyzed using the χ^2^ test of independence, pairwise comparisons, and Cramér’s coefficient. The results were described in terms of frequency and relative frequency (percentage). The observed differences and dependency were considered statistically significant if the calculated *P*-value was below 0.05.

## Results

### The threat posed by the COVID-19 pandemic

Responses to the statement “*The COVID-19 pandemic posed a threat to the life and safety of my colleagues and I*” were distributed as follows: 33 (17.27%) physicians disagreed or neither agreed nor disagreed; 83 (43.5%) physicians agreed; and 75 (39.3%) physicians strongly agreed. Statistically significant differences among the three groups were noted in the age (*P* = 0.046) and work experience (*P* = 0.039) variables. Pairwise comparisons of the Kruskal–Wallis criterion revealed that both characteristics were only statistically significantly different between two groups: those who disagree or neither agree nor disagree and those who strongly agree (Table 1).

**Table 1.** The assessment of the threats posed by the COVID-19 pandemic as perceived by physicians

Characteristic	The COVID-19 pandemic poses a threat to mine and my colleagues’ life and safety			*P*-value*
	Strongly disagree/neither agree nor disagree	Agree	Strongly agree	
Age (years)	51 (35.5–58.5)^a^	53 (37–66)	59 (47–68)^a^	0.046 a 0.04
Work experience (years)	25 (7–33)^b^	22 (9–41)	34 (20–40)^b^	0.039 b 0.042

*Note*. Data presented as a median (Q_1_–Q_3_). *The Kruskal–Wallis criterion; a–b pairwise comparisons with Bonferroni correction.

As a result, H1 is confirmed: physicians’ perceptions of the threat posed by the COVID-19 pandemic depend on their sociodemographic characteristics – namely, age and work experience.

### Overall work satisfaction among physicians

Based on responses to the statement “*My work satisfaction decreased during the COVID-19 pandemic*”, physicians working in family physician teams were divided into three groups: disagree or neither agree nor disagree (*n* = 52, 27.23%); agree (*n* = 76, 39.8%); and strongly agree (*n* = 63, 33%). These groups demonstrated a statistically significant difference in age (*P* = 0.001), work experience (*P* < 0.001), and duration of employment (*P* = 0.012) (Table 2). Pairwise comparisons of the Kruskal–Wallis criterion revealed statistically significant differences in age, work experience, and duration of employment between all three groups. Sample characteristics showed a higher proportion of younger, less experienced respondents and those who had been employed for a shorter time clustered in the *strongly agree* or *neither agree nor disagree* categories. Conversely, no difference between the same characteristics was observed among physicians who agreed or strongly agreed with the statement.

**Table 2.** Work satisfaction among physicians during the COVID-19 pandemic

Characteristic	My work satisfaction has decreased during the COVID-19 pandemic			*P*-value*
	Strongly disagree/neither agree nor disagree	Agree	Strongly agree	
Age (years)	42.5 (34–58)^a,b^	59 (50–59)^a^	58 (42–67)^b^	0.001 a 0.002; b 0.007
Work experience (years)	15 (5–30.75)^c,d^	35 (12–39.75)^c^	32 (15–42)^d^	<0.001 c 0.001; d 0.001
Duration of employment (years)	5.5 (5–18.75)^e,f^	18 (4–33.75)^e^	17 (5–33)^f^	0.012 e 0.025; f 0.025

*Note*. Data were presented as a median (Q_1_–Q_3_). *The Kruskal–Wallis criterion; a–f pairwise comparisons with Bonferroni correction.

The study outcome confirmed H2, which postulated that the overall work satisfaction of physicians working in family physician teams decreased during the COVID-19 pandemic.

### Patient satisfaction with service provision

The statement “*The satisfaction of my patients with the services provided by their family medical institution decreased during the COVID-19 pandemic*” yielded the following responses: 64 (33.51%) physicians disagreed or neither agreed nor disagreed; 83 (43.5%) agreed; and 44 (23%) strongly agreed. The only statistically significant difference between these groups was observed in the work experience characteristic (quartiles of 24.5 (7.25–36.75), 35 (14–42), and 21 (7.5–40), respectively; *P* = 0.037). Pairwise comparison of the Kruskal–Wallis criterion yielded a statistically significant difference in work experience between the *agree* and *disagree or neither agree nor disagree* groups (*P* = 0.018). H3 was thus confirmed, suggesting that patient satisfaction with the family medicine services provided decreased during the pandemic.

### Access to services

The statement “*My patients experienced reduced access to family medicine services during the COVID-19 pandemic*” was met with the following responses: 57 (29.84%) physicians disagreed or neither agreed nor disagreed; 91 (47.6%) physicians agreed; and 43 (22.5%) strongly agreed. Statistically significant differences in age (*P* = 0.004), work experience (*P* = 0.003), and duration of employment (*P* = 0.012) were observed among these groups (Table 3). Pairwise comparison of the Kruskal–Wallis criteria demonstrated a statistically significant difference in terms of these characteristics among two groups: those who agreed and those who disagreed or neither agreed nor disagreed with the statement.

**Table 3.** Patient access to services during the COVID-19 pandemic

Characteristic	My patients experienced reduced access to family medicine services during the COVID-19 pandemic			*P*-value*
	Strongly disagree/neither agree nor disagree	Agree	Strongly agree	
Age (years)	50 (34.5–59.5)^a^	61 (50–68)^a^	51 (37–64)	0.004 a 0.005
Work experience (years)	20 (6.5–34.5)^b^	35 (20–41)^b^	23 (8–40)	0.003 b 0.002
Duration of employment (years)	8 (3–26)^c^	20 (7–35)^c^	10 (2–30)	0.012 c 0.033

*The Kruskal–Wallis criterion; a–c pairwise comparisons with Bonferroni correction.

These findings showed that patients experienced reduced access to services during the pandemic, and H4 was thus confirmed: physicians perceived a decrease in their patients’ level of access to services during the pandemic.

### The need for new tools in the provision of consultations

The majority of respondents from both urban- and rural-based PHCIs indicated that new tools are needed to facilitate the provision of standard consultation services during the COVID-19 pandemic (Table 4). However, agreement was not homogenous: statistically significantly more urban-based respondents agreed rather than strongly agreed with this statement (97.3% and 80.2%, respectively).

**Table 4.** The need for new tools for patient consultations during the COVID-19 pandemic

Characteristic	Standard consultations require new tools during the COVID-19		
	Strongly disagree/neither agree nor disagree	Agree	Strongly agree
City-based PHCI	29 (90.6)	71 (97.3)^a^	69 (80.2)^a^
Rural-based PHCI	3 (9.4)	2 (2.7)^b^	17 (19.8)^b^

*Note. χ* ^2^ = 11.406; *r*_cr_ = 0.244; *P* = 0.003; a, b pairwise comparisons *P* < 0.05.

These results confirmed H5, which stated that the perceived need for new tools during the pandemic was dependent on the location of the respondent’s workplace (urban/rural setting).

## Discussion

The majority of health care institutions failed to meet the challenges posed by the COVID-19 pandemic with quick and decisive action (Alketbi *et al*., 2022). Generally, crisis management utilizes the following tools: information technologies, strategic planning, communication, media, information management, leadership, and effective governance at the national level (Hazaa *et al*., 2021). Some researchers found that crisis management in the uncertainty of the COVID-19 pandemic could be positively impacted by employee training, leadership, an effective organizational strategy, organizational structure, and organizational culture (Alketbi *et al*., 2022). A link has been established between competencies in crisis management and team-level activities pertaining to information sharing, teamwork, and the identification of crises (Jankelová *et al*., 2021). In a crisis, institutional values and employee satisfaction facilitate effective service provision and employee well-being (Alketbi *et al*., 2022). Over the course of this study, it has been determined that physicians’ age and work experience affect their perception of the threat posed by the COVID-19 pandemic to their life and safety. Leadership, communication, public image, innovation, and a strong team led by a good manager are critical to the successful management of a crisis situation (Dwiedienawati *et al*., 2021). This study found that the majority of respondents supported the use of innovative technologies to augment standard consultation experience. Elsewhere, family physician teams expressed a greater level of overall work satisfaction than their single-practice colleagues (Werdecker and Esch, 2021). Although female physicians demonstrate a higher level of work satisfaction than males (Werdecker and Esch, 2021), this study found that satisfaction was dependent on the physician’s age, work experience, and duration of employment in their current institution. Furthermore, patient satisfaction is conditional on positive patient–physician interactions and plays a key role in building long-term relationships (Rajić *et al*., 2021). This study found that work experience determined how respondents perceived patient satisfaction with the services provided by family medical institutions during the COVID-19 pandemic. Similarly, physicians’ age, work experience, and duration of employment in their current institution impacted their perception of patients’ access to services during the pandemic.

### Limitations

This study yielded a mass of scientific data regarding physicians’: perception of their individual satisfaction and that of their patients; perceived level of threat posed by the COVID-19 pandemic; perception of patient access to services; and views pertaining to the use of innovative technologies for consultation management. The key limitation of this study concerns the sample population, which encompassed exclusively primary-level health care workers (physicians working in family physician teams). More insight could be gained from hospital-based physicians and those involved in emergency services. Furthermore, the anonymous survey was conducted within a single country, and a comparison with equivalent data from other countries could provide an interesting perspective.

### Implications for practice

In an effort to cope with the challenges of crisis management during the COVID-19 pandemic, physicians working in family physician teams must be afforded access to proper mental health care services. The pandemic prompted a fall in overall work satisfaction among physicians; therefore, additional training for physicians regarding patient interactions would be beneficial. Patients were less satisfied with services provided during the pandemic; this could be mitigated by ensuring effective patient–physician communication. New tools to aid with patient consultations (e.g., telemedicine, psychological support, small-group workshops, training, etc.) must be introduced and effectively applied in practice during crisis situations in the health care sector.

## Conclusions

- Physicians should be afforded professional mental health care services and support, beyond the simple provision of PPE to protect against infection.
- Crisis management necessitates new tools for providing consultations to patients.
